# New Frontiers of Biomarkers in Metastatic Colorectal Cancer: Potential and Critical Issues

**DOI:** 10.3390/ijms26115268

**Published:** 2025-05-30

**Authors:** Bianca Medici, Stefania Benatti, Massimo Dominici, Fabio Gelsomino

**Affiliations:** Department of Medical and Surgical Sciences for Children and Adults, Division of Oncology, University Hospital of Modena, 41124 Modena, Italy; bia.medici31@gmail.com (B.M.); stefania.benatti@unimore.it (S.B.); massimo.dominici@unimore.it (M.D.)

**Keywords:** metastatic colorectal cancer, multi-omics sciences, personalized therapies

## Abstract

Metastatic colorectal cancer (mCRC) remains a major cause of cancer-related mortality worldwide and requires the development of new biomarkers to improve patient management. Traditional markers, such as RAS mutations and microsatellite instability (MSI), have revolutionized therapeutic decisions, but emerging evidence underlines the importance of integrating multi-omics sciences for a deeper understanding of tumor biology and therapeutic resistance. Although these omics technologies hold great promise for the advancement of precision oncology, significant challenges remain. However, the integration of multi-omics data is opening the way to more accurate diagnostics, personalized therapies, and improved outcomes for mCRC patients. This review provides an in-depth description of the various omics sciences and explores their advantages and critical issues.

## 1. Introduction

Colorectal cancer (CRC) remains a leading cause of cancer-related mortality worldwide, with its incidence expected to rise significantly in the coming years. This complex and heterogeneous disease is driven by multiple molecular alterations, necessitating precise biomarkers for risk assessment, treatment decisions, and disease monitoring. Current clinical practice employs biomarkers such as RAS mutations, microsatellite instability (MSI), liquid biopsy, and circulating tumor DNA (ct DNA) for guiding treatment strategies [1].

Prognostic markers are those that predict clinical outcomes without treatment, while predictive markers stratify patients based on their response to a specific treatment. Ideally, a biomarker possesses both prognostic and predictive value. Validation of predictive markers requires well-designed randomized controlled trials [1].

Emerging biomarkers, including tumor mutational burden (TMB), HER2 amplification, and novel molecular signatures are being investigated for their potential to improve treatment stratification [2].

Molecular profiling offers a more precise, patient-specific approach, allowing for better risk assessment, therapy selection, and surveillance strategies. However, for widespread clinical integration, standardization, cost reduction, and validation of biomarkers remain crucial challenges [3].

In recent years, the advent of omics sciences including genomics, transcriptomics, proteomics, and metabolomics has revolutionized our understanding of the molecular landscape of metastatic CRC (mCRC). These approaches provide comprehensive insights into tumor biology, enabling the identification of novel biomarkers, therapeutic targets, and mechanisms of drug resistance.

This review explores the latest applications of omics sciences in mCRC, evaluating positives and negatives of new biomarkers that are appearing in clinical practice, highlighting their role in precision oncology and in developing personalized therapeutics.

## 2. Omics Sciences

Cancer is a multi-factorial disease driven by complex interactions between internal and environmental factors, leading to diverse molecular alterations. This heterogeneity necessitates a shift from one-size-fits-all treatments to personalized approaches [4].

Traditional biomarkers (e.g., PD-L1, tumor mutational burden) do not work for all patients. Multi-omics enables new predictive models based on immune cell dynamics, microbiome interactions, and metabolic changes [5].

Multi-omics approaches integrate molecular data across different biological levels to identify novel biomarkers, classify CRC subtypes, and improve early detection and personalized treatment strategies. Each omics field contributes uniquely, but a single-omics approach is insufficient (Table 1).

Multi-omics integration is essential for a holistic understanding of cancer and analyzing different levels of biological information, from DNA to RNA and proteins, providing a comprehensive view of CRC development [4,6,7,8].

Furthermore, immunomics and microbiomics demonstrate that cancer is not an isolated cellular phenomenon but an interaction between the tumor, the immune system, and external factors. This perspective challenges conventional treatment approaches, advocating for a more holistic and patient-specific strategy. As multi-omics tools become more refined, they will enhance the accuracy of cancer prognosis and treatment response prediction, making personalized medicine a practical reality [9].

Multi-omics can predict patient responses to chemo- and immunotherapy, leading to more effective personalized treatments and the integration of various data sources enables the identification of reliable diagnostic and prognostic markers. However, these potent techniques have limitations; for example, genome sequencing cannot explain phenotypic outcomes alone, whereas epigenomic changes need transcriptomic data for functional interpretation and mRNA levels alone do not always correlate with protein expression [10].

The development of these technologies is rapidly advancing, with costs decreasing and efficacy improving and they are being applied across various fields, including in cancer research for analyzing complex tumor structures, identifying subtypes, and exploring pathways involved in tumor development [11].

Traditional single-omics studies (genomics, epigenomics, transcriptomics, proteomics, metabolomics) provide partial insights into cancer mechanisms but lack a full causal relationship between molecular alterations and phenotypic changes. On the other hand, multi-omics integration allows a systems biology approach, linking molecular events at different levels to cancer hallmarks like metastasis, immune evasion, and therapy resistance [10].

The multi-omics model represents a paradigm change in cancer research by moving from a single-gene or single-protein perspective to a systems biology approach. Traditional cancer research often focuses on isolated genetic mutations, but this view fails to capture the complexity of cancer progression and resistance mechanisms [12].

The integration of multiple omics layers allows for a more holistic understanding, leading to:

- More accurate diagnostics: Combining genomic and epigenomic data enhances the identification of cancer subtypes.
- Personalized treatment strategies: Multi-omics enables tailored therapies by identifying patient-specific molecular targets.
- Improved drug development: Systems biology approaches help predict drug responses and resistance patterns.

**Figure 1:**
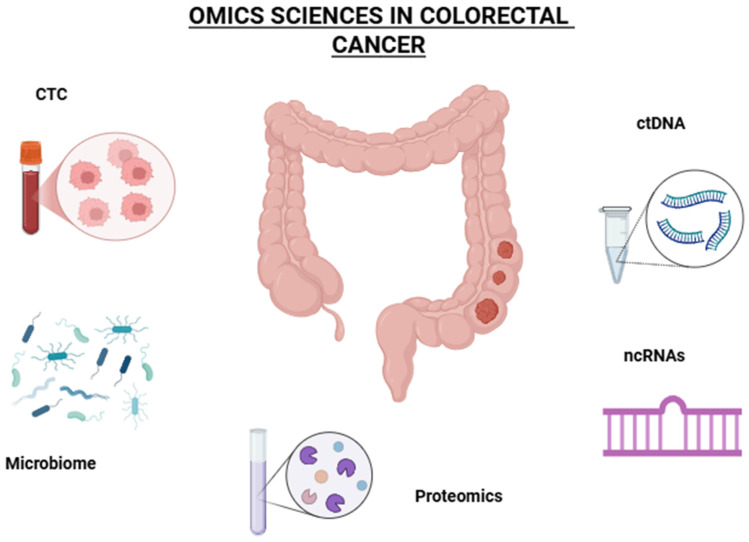
Omics sciences in colorectal cancer. CTC: circulating tumor cells; ctDNA: circulating tumor DNA; ncRNAs: non-coding RNAs. The figure was created by BioRender online software (BioRender.com accessed on 7 May 2025).

In conclusion, by integrating data from these omics technologies, researchers can have a deeper understanding of CRC pathogenesis, identify potential biomarkers for early diagnosis and develop more effective treatment strategies. This holistic approach holds promise for improving CRC patient outcomes and advancing personalized medicine [10,13] (Figure 1).

### 2.1. Genomics

Genomics in oncology studies the entire genetic background (DNA) of cancer cells to identify genetic alterations that are responsible for the onset, progression, and response to cancer treatments. Using next-generation sequencing (NGS) and other high-processing technologies, it has been possible to map mutations, gene copy number variations, epigenetic modifications, and gene expression patterns that drive oncogenesis. In CRC, these analyses identified crucial signaling pathways, such as WNT, RAS-MAPK, PI3K, TGF-β, and p53, and recurrent mutations in key genes such as APC, KRAS, TP53, and PIK3CA [3,14].

In addition to somatic mutations, germline predisposition and epigenetic modifications also play a significant role in the pathogenesis of CRC, influencing its susceptibility and prognosis. Indeed, CRC is a heterogeneous disease, characterized by chromosomal instability (CIN), microsatellite instability (MSI), and CpG islet methylation phenotype (CIMP). Although primary and metastatic tumors share similar mutational profiles, subclonal evolution and influences of the tumor microenvironment may result in different behaviors, especially in the metastatic phase. In particular, specific alterations such as increased frequency of TP53 mutations or reduced incidence of BRAF mutations in metastases suggest a possible role of treatment-induced selection or metastatic adaptation [15]. In this context, performing metastatic biopsies could offer clinically relevant information, especially in patients with multiple tumors or prior treatment [15,16,17].

The Cancer Genome Atlas has categorized CRC into mismatch repair-deficient/microsatellite instability (dMMR/MSI) and mismatch repair proficient/microsatellite stability (pMMR/MSS) subtypes. While MSI tumors, characterized by high mutational burdens and neoantigens formation, respond well to immune checkpoint inhibitors (ICIs), the majority of CRC cases (MSS tumors) remain resistant due to low immunogenicity and an immunosuppressive microenvironment. Recent findings indicate that a subset of MSS tumors with hypermutated phenotypes, such as those harboring POLE mutations, may also benefit from immunotherapy [14,18].

Finally, the mutational profile of CRC varies according to tumor site and age of onset. Early-onset CRC (EOCRC, <50 years) show significant differences from late-onset CRC (LOCRC), especially at the level of chromosomal alterations and frequency of mutations in KRAS and BRAF V600E. In addition, metastatic EOCRC forms appear to possess distinctive molecular features, with potential implications for personalized therapeutic approaches [14].

#### 2.1.1. MMR Genes

Defects in MMR genes, leading to MSI, are both prognostic and predictive. MSI tumors often exhibit distinct characteristics and, while associated with better prognosis in early stages, may show less benefit from 5-fluorouracil (5-FU) based chemotherapy. The mechanism of this potential resistance is still a matter of debate, given the complexity of interpreting retrospective data and the need for prospective trials [19,20,21].

MSI, present in both hereditary (Lynch syndrome) and sporadic CRCs, is characterized by MMR gene mutations. The proportion of MSI/dMMR tumors in EOCRC varies. While some studies indicate most EOCRC is MSS, others report higher MSI frequencies in younger patients. BRAF mutations are less common in EOCRC MSI/dMMR tumors, likely due to the predominance of Lynch syndrome-related MSI in this group. MSI/dMMR EOCRC may have a poorer prognosis despite being a positive prognostic factor in earlier stages [14].

The prognostic value of 18q LOH is controversial. While some studies suggest an association with worse outcomes, others, particularly those accounting for MSI status, have failed to confirm this. The ECOG 5202 trial is designed to address this question, though its design has been criticized for not directly comparing adjuvant therapy vs. no therapy in MSI patients [19,21].

#### 2.1.2. RAS and BRAF

RAS mutations are common in CRC and have been extensively studied [19,21]. KRAS mutations are present in approximately 35–45% of cases, whereas NRAS mutations occur in about 3–5% of CRCs [22,23,24]. RAS mutations are negative predictive biomarkers for response to anti-EGFR monoclonal antibodies, such as cetuximab and panitumumab. The presence of RAS mutations is associated with poorer prognosis and may correlate with a predilection for lung rather than liver metastases [25].

BRAF mutations, often found in MSI and CIMP positive tumors, are also being investigated. Many studies unequivocally demonstrated their association with worse prognosis, particularly in MSS tumors. BRAF mutations also predict resistance to anti-EGFR therapies, though data are less robust as compared to RAS [19].

#### 2.1.3. PI3K/AKT Pathway

PIK3CA mutations are associated with constitutive activation of the PI3K/AKT pathway. Some studies suggest a prognostic role, particularly in KRAS wild-type tumors and a predictive role for resistance to cetuximab. Furthermore, PTEN is another component of the PI3K/AKT pathway. Its loss has been linked to worse outcomes in some studies, but the data are inconsistent. Its predictive value for anti-EGFR therapy is also being explored [19].

#### 2.1.4. Conclusions

Globally, genomics has transformed the understanding of colorectal cancer by highlighting its molecular complexity and interindividual variability. Genetic and epigenetic information not only enables more accurate classification of the disease, but also guides the identification of therapeutic targets and prediction of response to treatments, including immunotherapy. However, integration of these data into clinical practice requires further study and validation, particularly to define the role of metastatic biopsies and molecular features in early-onset cancers.

### 2.2. Circulating Tumor DNA

Circulating tumor DNA (ctDNA) refers to fragments of DNA that are shed from tumor cells into the bloodstream. These fragments are typically short, comprising fewer than 200 nucleotides. As tumors grow, cells undergo apoptosis or necrosis, releasing their contents, including DNA, into the blood. ctDNA carries genetic mutations specific to the tumor, making it a valuable biomarker for non-invasive cancer diagnostics, monitoring and prognosis.

ctDNA analysis is a promising tool for real-time monitoring of mCRC, with strong diagnostic and prognostic value. While it cannot fully replace tissue biopsy, its advantages in detecting resistance mechanisms and guiding personalized treatment strategies make it a valuable addition to clinical practice [26].

ctDNA detects genetic alterations, improving patient stratification for targeted therapies. Indeed, ctDNA levels correlate with early tumor response and can predict resistance to anti-EGFR, anti-HER2, and BRAF-targeted therapies, helping to identify patients who may benefit from reintroducing anti-EGFR therapy after resistant clones have disappeared. On the other hand, false negatives may occur in tumors with low ctDNA shedding, particularly in certain metastatic sites (e.g., lungs, peritoneal); moreover, there are no widely accepted cutoffs for ctDNA-guided treatment decisions [27,28].

In adjuvant setting, ctDNA identifies high-risk patients post-surgery who may benefit from adjuvant therapy. Analysis of ctDNA for detecting minimal residual disease can identify those patients who are at higher risk of relapse [29,30].

In the DYNAMIC trial, a ctDNA-guided approach to the treatment of stage II colon cancer reduced adjuvant chemotherapy use without compromising recurrence-free survival [31].

CtDNA-based post-surgical detection of molecular residual disease is known to be predictive of a high risk of recurrence, as demonstrated in the BESPOKE trial [32].

Moreover, analysis from the GALAXY trial shows that patients with CRC with sustained ctDNA clearance post adjuvant chemotherapy are less likely to experience recurrence compared with those who have transient or no ctDNA clearance [33].

ctDNA is also investigated in metastatic setting and has potential in detecting genomic alterations, predicting treatment responses and monitoring resistance to targeted therapies and offering a real-time and minimally invasive alternative for assessing genetic mutations. Mutations in RAS, BRAF, ERBB2, and MET influence resistance to anti-EGFR therapy and ctDNA can track acquired mutations like KRAS and EGFR mutations that signal resistance [34].

One of the most pivotal trials is the CHRONOS Trial, a phase 2, single-arm clinical trial, focused on mCRC patients with RAS wild-type tumors who initially benefit from anti-EGFR monoclonal antibody Panitumumab. The CHRONOS trial aimed to determine whether ctDNA analysis could guide rechallenge with Panitumumab by selecting patients without detectable resistance mutations and it is the first to use ctDNA prospectively to guide anti-EGFR rechallenge in mCRC. While 30% of patients had a partial response, 63% achieved disease control (response + stable disease). This study highlights liquid biopsy as a promising tool for precision oncology, allowing real-time monitoring of tumor evolution and treatment resistance in mCRC [35].

Other studies have also shown that ctDNA can detect the presence of target mutations, such as KRAS in metastatic setting. Plasma ctDNA testing effectively tracked treatment response, detecting emerging RAS mutations linked to anti-EGFR resistance and eliminating the need for repeated invasive tumor biopsies. The concordance also seems to be 93% between plasma and tissue RAS status. Discrepancies were linked to tumor heterogeneity, the site of metastasis, and prior treatments; indeed, sensitivity may vary based on tumor burden and metastatic site [26].

ctDNA demonstrated a stronger correlation with tumor burden than carcinoembryonic antigen (CEA), the standard blood biomarker. A study investigated the predictive power of ctDNA in assessing treatment response earlier than conventional imaging methods (RECIST criteria). It involved 53 metastatic CRC (mCRC) patients receiving first-line chemotherapy, analyzing their ctDNA levels before treatment, three days after treatment and before the second cycle of chemotherapy. ctDNA was detectable in 92.3% of patients and its early reduction correlated with radiological responses at 8–10 weeks. A ≥10-fold drop in ctDNA before the second cycle was associated with improved progression-free survival (PFS). The study suggests that ctDNA could serve as an early biomarker to guide treatment decisions, potentially reducing exposure to ineffective therapies [36].

The effectiveness of next-generation sequencing (NGS) of ctDNA in mCRC has been evaluated to assess whether a moderate-sized 12-gene NGS panel can be used for ctDNA-based monitoring of genetic variants and treatment response in mCRC. There was a high concordance (85%) between tumor tissue and plasma ctDNA results. ctDNA levels were significantly correlated with tumor shrinkage (*p* = 0.041). A ctDNA post-treatment decrease of >80% was associated with improved PFS and better objective response rates (OR = 0.026; *p* = 0.007). The study demonstrated that NGS-based ctDNA monitoring is a viable method for evaluating disease progression and drug resistance, even more accurate than traditional biomarkers like CEA [37].

The ctDNA was also investigated in the setting of metastatic resected disease. A study evaluated the prognostic impact of ctDNA in patients undergoing curative surgery for colorectal cancer liver metastases. Plasma samples were collected before and after surgery, during adjuvant chemotherapy and in follow-up for ctDNA analysis. ctDNA was detectable in 85% of patients before treatment. About 24% of patients had detectable ctDNA after surgery, and they had an 83% recurrence rate compared to 31% in ctDNA-negative patients. Patients with persistently detectable ctDNA after adjuvant chemotherapy had 100% recurrence risk. Patients with undetectable post-treatment ctDNA had a 5-year recurrence-free survival rate of 75.6%, whereas those with detectable ctDNA had 0% recurrence free survival (RFS) [38].

Moreover, the Unicancer PRODIGE-14 Trial investigated the role of ctDNA and circulating tumor cells (CTCs) in patients with mCRC undergoing systemic therapy before liver metastasis resection. A total of 153 mCRC patients with potentially resectable liver metastasis received first-line chemotherapy (doublet or triplet) with targeted therapy (bevacizumab or cetuximab based on RAS status). Baseline ctDNA detection was 91% sensitive in identifying KRAS mutations compared to tissue biopsy. ctDNA levels decreased significantly during treatment, but persistent detection after 4 weeks correlated with lower liver metastasis resection rates (*p* = 0.01) and shorter overall survival (*p* < 0.001). Patients with undetectable ctDNA pre-surgery had significantly longer post-operative survival (HR = 31, *p* < 0.001). CTC counts decreased significantly during therapy, but persistent detection at 4 weeks indicated shorter survival (HR = 10.9, *p* < 0.001). This study reinforces the potential of ctDNA and CTCs as powerful prognostic and predictive biomarkers in mCRC. However, further standardization and validation are required before its introduction in routine clinical practice [39].

Another study investigates the role of ctDNA as a biomarker for predicting recurrence in metastatic colorectal cancer patients who undergo resection of metastases. ctDNA assay was used to detect molecular residual disease (MRD). MRD-positive patients had significantly shorter OS (HR = 16.0, *p* < 0.001) and patients with persistent ctDNA after adjuvant therapy had 100% recurrence risk. ctDNA had higher sensitivity (84.6%) compared to carcinoembryonic antigen (CEA) (46%) for detecting recurrence. This study suggests a possible personalized treatment decision: MRD-negative patients could avoid unnecessary chemotherapy, reducing toxicity; on the other hand, MRD-positive patients might benefit from escalated therapy or clinical trials. This study strongly supports ctDNA-based MRD detection as a powerful prognostic tool for identifying mCRC patients at risk of relapse [40].

Further field of research is ctDNA methylation. Indeed, methylated ctDNA (meth-ctDNA) may serve as an early predictor of treatment benefit in patients receiving first-line chemotherapy. In a study, meth-ctDNA was analyzed before treatment (T0) and after the first cycle (T1). In particular, patients were classified into two groups, low ctDNA (meth-ctDNA levels dropped to or near zero) and high ctDNA (meth-ctDNA remained stable, increased, or only slightly decreased). A low meth-ctDNA level after one cycle correlates with better survival outcomes, so meth-ctDNA seem to be a promising biomarker for early identification of treatment benefit in mCRC [41].

In conclusion, ctDNA appears to be a very good prognostic and predictive marker in both adjuvant and metastatic settings; it also appears to have better sensitivity than conventionally used markers in clinical practice. On the other hand, further studies are needed to overcome some issues arising from the fact that not all disease sites release ctDNA equally, and not all the methods used for analysis are equally predisposed. An additional problem is the question of the cost to be incurred to use such a biomarker.

### 2.3. Transcriptomic

Transcriptomics is the study of the complete set of RNAs transcribed in a cell at a given time and includes both coding (mRNA) and non-coding RNAs, including microRNA (miRNA) and long non-coding RNA (lncRNA). MiRNAs are small regulatory RNAs (~21–25 nucleotides) that modulate gene expression by binding to target mRNAs and determining their degradation or inhibiting their translation [42]. lncRNAs are RNAs over 200 nucleotides long that do not encode proteins but regulate gene expression at various levels, from epigenetics to transcription and mRNA processing [43].

Using techniques such as RNA sequencing (RNA-seq) and microarrays, transcriptomic science helps researchers [44]:

- Identify gene expression patterns that distinguish different cancer types and subtypes;
- Discover biomarkers for diagnosis, prognosis and treatment response;
- Understand tumor heterogeneity and the role of the tumor microenvironment;
- Explore mechanisms of drug resistance and metastasis.

This field plays a crucial role in precision oncology, enabling personalized treatment strategies based on an individual patient’s tumor transcriptome [45].

Transcriptomics analysis allows us to study not only the tumor itself, but also the environment around neoplasm, particularly the immune microenvironment in colorectal cancer. In fact, it is becoming increasingly understood that the peritumoral environment has an important effect on both the response or resistance to treatments and the course of the neoplasm.

An analysis explored the tumor immune microenvironment in colorectal cancer liver metastases using single-cell RNA sequencing and identified 12 cell clusters corresponding to six main cell types: cancer cells, T cells, myeloid cells, endothelial cells, fibroblasts and B cells. A key finding was the high heterogeneity of the immune microenvironment, particularly the enrichment of granulocytes in tumor samples. Researchers discovered 445 deregulated genes across different cell clusters, organizing them into six gene modules. Among them, 93 genes were specifically deregulated in tumor-infiltrating immune cells, showing a correlation with patient survival rates. The study highlights the IL-17 signaling pathway’s role in granulocytes, potentially influencing CRC metastasis. Additionally, an abnormal ferroptosis-mediated cell death mechanism in granulocytes was proposed as a factor in their increased presence within tumors. Overall, this research provides valuable insights into the immune landscape of CRC metastases, paving the way for new therapeutic strategies targeting immune cell interactions within the tumor microenvironment [46].

Another study investigates the immune microenvironment in colorectal cancer liver metastases using spatial transcriptomics. Researchers analyzed tumor samples from patients who underwent synchronous resection of primary colorectal cancer and liver metastases. The goal was to identify histological and molecular markers that could predict patient outcomes. Findings show that tumors with high immune infiltration at the invasive edge had better survival rates, while those with increased regulatory T cells and neutrophils at the tumor center had worse outcomes. The research highlights significant heterogeneity between and within tumors, demonstrating that bulk transcriptomic analysis can be confounded by stromal content. Spatial transcriptomics provided more precise insights into the tumor immune landscape, suggesting potential biomarkers and therapeutic targets [47].

Moreover, genomic and transcriptomic changes influence therapeutic responses in metastatic colorectal cancer. Tumors with a higher number of subclones in their primary stage exhibited more dynamic changes during metastasis. The study also found that distant metastases (e.g., to the liver) and loco-regional metastases (e.g., to lymph nodes) followed different evolutionary paths [48].

Through transcriptomics analysis, differences between primary tumor and metastatic site were analyzed. Variability in transcriptomic analysis is also present within the same metastases [49].

A study examines the transcriptomic differences between primary colorectal adenocarcinomas and their distant metastases, aiming to identify unique gene expression patterns that could help predict metastasis risk and inform targeted therapies. The study found that metastases exhibited reduced epithelial–mesenchymal transition (EMT) but increased activation of MYC target genes and DNA repair pathways. The most differentially expressed genes between primary and metastatic tumors were FBN2 and MMP3. Researchers identified two metastatic subtypes—EMT inflammatory and proliferative—which were distinct from the widely recognized consensus molecular subtype (CMS) 3 of colorectal cancer. These findings highlight a specific gene-expression signature for metastatic colorectal cancer, providing insights that could guide the development of metastasis-targeted treatments [50].

Another analysis investigates the genomic and transcriptomic characteristics of primary colorectal and liver carcinomas and their matched distant metastases to identify key metastasis-related genes. Primary tumors and their metastatic counterparts share highly similar gene expression profiles, suggesting that metastatic potential is already present in the primary tumor rather than acquired through additional mutations. However, liver and colorectal metastases exhibited distinct gene expression patterns, highlighting organ-specific molecular adaptations. The study also found that liver metastases displayed higher genomic instability compared to primary tumors. Interestingly, metastasis-associated gene signatures were different across organ sites, but all shared prognostic value, indicating their potential use in predicting patient outcomes [51].

Furthermore, there is a high similarity in gene expression between primary tumors and their corresponding liver metastases, suggesting that metastatic potential is already present in the primary tumor rather than acquired later. A major discovery was the identification of fusion transcripts, including a novel RNF43-SUPT4H1 fusion gene that was frequently present in primary tumors. Functional studies revealed that silencing this fusion gene reduced tumor cell proliferation, suggesting its role in colorectal cancer progression. Overall, this study highlights the molecular similarities between primary and metastatic colorectal tumors, while also identifying fusion genes as potential therapeutic targets [52].

Another use of the transcriptomics technique is to study mechanisms of resistance to treatments.

The mechanisms of resistance to anti-EGFR therapy, specifically cetuximab, have been investigated in metastatic colorectal cancer. Researchers analyzed genomic and transcriptomic data from 35 patients with RAS wild-type CRC who were treated with cetuximab. Resistance can be driven by both genetic and non-genetic mechanisms. Genetic alterations, such as NF1 loss and non-canonical mutations in KRAS and BRAF, were linked to primary resistance. However, in 64% of cases that developed acquired resistance, no genetic mutations were detected. Instead, resistance was associated with changes in the tumor microenvironment, particularly a shift towards a fibroblast- and growth factor-rich subtype, which promotes drug resistance through stromal remodeling. Interestingly, cetuximab treatment was found to increase immune infiltration in tumors, leading to the upregulation of immune checkpoints like PD-L1 and LAG3. This suggests that combining cetuximab with immunotherapy could be a promising strategy for overcoming resistance [53].

Transcriptomics has also been studied to find new prognostic gene markers for colorectal disease. Researchers compiled and analyzed a dataset of 1,273 colorectal cancer samples, integrating transcriptomic profiles with survival information. Their approach identified two sets of genes: those upregulated in patients with poor prognosis and those linked to better survival outcomes. Among the most significant survival markers for poor prognosis were DCBLD2, PTPN14, LAMP5, and TM4SF1, while genes like EPHB2 and DUS1L were associated with better survival rates [54].

In conclusion, transcriptomics represents a fundamental tool for understanding the biology of colorectal cancer in all its developmental stages, from diagnosis to metastatic progression and response to treatment. By analyzing the transcriptome, the complexity of the tumor microenvironment, inter- and intra-tumor variability, and the existence of distinctive molecular signatures in metastatic tumors have been highlighted. This information is crucial not only for identifying new prognostic and predictive biomarkers, but also for understanding the mechanisms of resistance to therapies, paving the way for increasingly targeted and personalized therapeutic strategies. From a precision oncology perspective, transcriptomics is thus confirmed as an essential pillar for improving the clinical management of colorectal cancer patients.

### 2.4. Proteomics

Proteomics in oncology refers to the large-scale study of proteins, their structures, functions and interactions within cancer cells and tumor microenvironments. It plays a crucial role in understanding cancer biology by identifying biomarkers for early diagnosis, prognosis and treatment response, as well as discovering potential therapeutic targets.

Using advanced techniques such as mass spectrometry, two-dimensional gel electrophoresis (2D-GE), and protein microarrays, proteomics allows researchers to identify protein biomarkers that could improve in early detection, prognosis assessment, treatment stratification, and disease monitoring. Despite the promising findings, the translation of proteomics-based biomarkers into clinical practice remains slow, primarily due to challenges in validation, standardization, and reproducibility across large patient cohorts [55,56,57,58].

As previously described, one of the first areas of application is the search for prognostic markers. A study investigated protein expression in CRC to identify biomarkers linked to prognosis. By comparing protein profiles of CRC tissues and normal colonic mucosa using two-dimensional gel electrophoresis and mass spectrometry, the researchers identified 45 proteins with at least 1.5 times increased expression in cancerous tissues. 14-3-3β emerged as a significant prognostic biomarker. Patients whose tumors lacked 14-3-3β expression had a better prognosis. A two-protein signature combining 14-3-3β and aldehyde dehydrogenase 1 (ALDH1) was identified as strongly predictive of patient outcomes, remaining independently prognostic even in multivariate analysis [59].

Overall, research explored the potential of proteomics in identifying biomarkers for colorectal liver metastases, which are the primary cause of death in patients with metastatic colorectal cancer. Liver resection is the primary curative treatment, but recurrence occurs in about 75% of patients, often within two years. Traditional clinicopathologic factors for predicting recurrence are inconsistent and lose significance over time. Proteomic biomarkers can identify patients at higher risk of developing liver metastases after primary CRC resection, detect the recurrence through liquid biopsy-based protein biomarkers earlier, and predict which patients will benefit from specific therapies, such as targeted treatments and chemotherapy [56,60].

It was investigated the molecular mechanisms behind colorectal cancer metastasis using advanced proteomic techniques. Although many studies have explored the transformation of normal cells into cancerous ones, the mechanisms that drive metastasis remain unclear. Since metastasis involves multiple factors, targeting only one or two proteins has proven ineffective in treatment. In metastatic cells there is the downregulation of β-catenin, a protein involved in cell adhesion, along with the upregulation of Calcyclin Binding Protein (CacyBP), which promotes β-catenin degradation. Further experiments confirmed that overexpression of CacyBP in CRC cells reduced adhesion and β-catenin levels, suggesting a role in metastasis [61].

Another study explored the molecular differences between primary and metastatic colorectal cancer using a multi-omics approach. The study analyzed 154 primary and 142 metastatic CRC samples, identifying six distinct proteomic subtypes. Metastatic tumors showed higher hypoxic stress, promoting epithelial-to-mesenchymal transition and metabolic adaptations, increased cancer stem cell characteristics and an alternative telomere lengthening phenotype, suggesting more aggressive tumor behavior. Finally, metastatic tumors were more immune-cold, with suppressed antigen presentation, potentially reducing the effectiveness of immunotherapy [62].

Exosomes are small vesicles secreted by cancer cells that facilitate cell-to-cell communication and can promote the formation of premetastatic niches, making them key players in cancer progression. A study compared the protein content of exosomes from primary and metastatic CRC cell lines using mass spectrometry-based proteomics, demonstrating that exosomes from metastatic CRC cells had a distinct protein profile enriched in metastatic factors (which are known to contribute to cancer cell migration and invasion), signal transduction proteins (which are involved in cell communication and tumor progression), and lipid raft components (which play a role in membrane signaling and cancer cell adaptation) [63].

With proteomics, extracellular vesicles have also been studied. The role of extracellular vehicles (EVs) in colorectal cancer progression and metastasis was investigated. Cancer cells actively release EVs, including exosomes and microvesicles, into their environment. These vesicles carry proteins, genetic material, and lipids that influence tumor progression, immune evasion, and drug resistance. Furthermore, using mass spectrometry-based proteomics, researchers compared EVs from primary CRC cells and their metastatic counterparts (Comparative Proteomic Analysis). EVs from primary tumors were enriched in cell adhesion proteins, reflecting their less invasive nature, whereas EVs from metastatic tumors contained proteins associated with cancer progression, immune suppression, and drug resistance [64].

Finally, among the proteins investigated, Stathmin-1 (STMN1) seems to be significantly upregulated in metastatic cells and was selected for further functional studies. Knockdown of STMN1 reduced cell migration, invasion, and adhesion, while overexpression led to increased metastatic potential. STMN1 seems to be a crucial factor in CRC metastasis, making it a promising biomarker for prognosis (higher STMN1 expression is linked to worse survival outcomes in CRC patients) and a potential therapeutic target [65].

Overall, proteomics represents a key tool for gaining insight into cancer biology, identifying diagnostic and prognostic biomarkers, and discovering new therapeutic targets. Despite challenges related to large-scale standardization and validation, advances in protein characterization of tumors and the tumor microenvironment are paving the way for more precise and personalized therapeutic strategies, especially in the context of colorectal cancer liver metastasis. Proteomic analysis has revealed key molecular differences between primary and metastatic tumors, helping to understand the mechanisms of tumor progression and treatment resistance.

### 2.5. Metabolomics

Metabolomics is the comprehensive study of small molecule metabolites within biological systems. In oncology, it is used to discover metabolites that can indicate the presence, stage, or aggressiveness of cancer, to study how cancer alters the body’s normal metabolic processes and to develop new tests for earlier cancer detection and better prediction of patient outcomes and to personalize treatment.

Metabolites related to cellular respiration, carbohydrate, lipid, protein, and nucleotide metabolism seem to be significantly altered in CRC. These altered metabolites may be also related to the prognosis, survival and recurrence of CRC [66].

Furthermore, researchers identified key metabolites related to redox balance, energy metabolism and amino acid, choline and nucleotide metabolism that change as CRC progresses. In addition, fecal studies suggested that changes in gut microbiota and short-chain fatty acids could serve as early CRC biomarkers [67].

A study explored how metabolomic profiling using proton nuclear magnetic resonance can help to identify patients with metastatic CRC and predict their survival outcomes. Metabolomic profiling could differentiate between healthy individuals and metastatic CRC patients with nearly 100% accuracy and patients with distinct metabolic profiles had significantly different survival rates. Differences in metabolite levels suggested metabolic shifts linked to cancer progression, inflammation, and energy metabolism [68].

Another approach investigated is gas chromatography–mass spectrometry on blood samples from healthy individuals, patients with adenomas (precancerous lesions) and CRC patients at different stages. A blood-based test for CRC screening could improve early detection rates and reduce reliance on invasive procedures like colonoscopy. In particular, researchers observed distinct metabolic alterations that correlated with CRC progression and metabolites related to energy metabolism, amino acid processing, and inflammation varied across different tumor stages [69].

Globally, metabolomics may improve the identification of patients with residual micrometastases after surgery. In a retrospective study, serum samples obtained after surgery from 94 elderly patients with early-stage CRC (65 relapse-free and 29 relapsed, after a median follow-up of 5 years) and from 75 elderly patients with metastatic CRC obtained before a new line of chemotherapy were retrospectively analyzed by proton nuclear magnetic resonance spectroscopy. Metabolomic classification was strongly associated with prognosis (*p*-value 0.0005, HR 3.64), independently of tumor stage [70].

Similarly, other studies evaluated whether metabolomics could be a tool to refine the risk stratification in elderly patients with early-stage colorectal cancer [71].

Moreover, it was hypothesized that metabolomic fingerprinting could identify patients with residual micrometastases after surgery who are at higher risk of relapse. The researchers used a combination of principal component analysis (PCA), canonical analysis (CA), and K-nearest neighbors (KNN) to develop a model that could discriminate between the metabolomic profiles of patients with relapse-free early CRC and those with metastatic CRC. The results showed that the PCA-CA-KNN model could successfully discriminate between relapse-free early CRC and metastatic CRC, with an accuracy of 70.0% using NOESY spectra. When the model was applied to patients with relapsed early CRC, it correctly predicted relapses in 69% of cases. Furthermore, the metabolomic classification was found to be strongly and independently associated with prognosis, with a *p*-value of 0.0005 and a hazard ratio of 3.64. The authors concluded that metabolomics could be a valuable tool for refining risk stratification in elderly patients with early CRC [72].

A study explored metabolomics and lipidomics to predict outcomes in 30 metastatic CRC patients who underwent liver metastasis resection after first-line conversion oxaliplatin-based chemotherapy plus bevacizumab. The authors found that pre-surgery levels of 3-hydroxybutyrate, cholesterol, phospholipids, triglycerides, and IL-6 were able to correctly classify patients by their DFS with good accuracy. Analysis of metabolomics, lipidomics, and cytokinomics has the potential to improve the prediction of outcomes in mCRC patients undergoing liver resection. However, the study had several limitations, including its retrospective design, small sample size and single-center setting [73].

Finally, Li et al. conducted a study to investigate the metabolic differences between circulating tumor cells (CTCs) and parental CT26 cells in CRC liver metastasis. The study found that the metabolic profiles of serum samples could differentiate between healthy controls and patients with metastatic CRC and identified five metabolites that were significantly different between the two groups. The authors concluded that the combined use of these five metabolites, along with other potential biomarkers, could improve the diagnosis and prognosis of metastatic CRC [74].

Metabolomics is thus emerging as a powerful approach to understand the metabolic alterations associated with colorectal cancer and to identify useful biomarkers to improve early diagnosis, prognostic stratification and personalization of treatment. Evidence shows that metabolic profiles reflect not only tumor progression and inflammatory status, but also the risk of recurrence and the presence of residual micrometastases, crucial aspects especially in elderly patients. Future clinical application of these data could facilitate more effective monitoring strategies and targeted treatments, significantly improving the management of metastatic colorectal cancer.

### 2.6. Immune Markers

Recent advances in the understanding of the tumor microenvironment have highlighted the crucial role of non-cancerous cells in colorectal cancer progression and metastasis. In particular, cancer-associated fibroblasts (CAFs), tumor-associated macrophages (TAMs), and tumor-infiltrating lymphocytes (TILs) have emerged as key players that can either promote or suppress tumor growth, depending on their phenotype and interactions with cancer cells.

CAFs come from different sources, such as resident fibroblasts and mesenchymal stem cells and are commonly found in the tumor microenvironment. They support CRC cell growth and survival by releasing growth factors like TGF-β, HGF, EGF, and FGF, which activate important signaling pathways such as MAPK and PI3K/AKT. CAFs also change the structure of the extracellular matrix and produce enzymes like MMPs, making it easier for cancer cells to invade and metastasize [75].

In addition, CAFs help form new blood vessels by producing VEGF, IL-6 and other molecules that promote angiogenesis. On a metabolic level, they provide energy to cancer cells through a process known as the “reverse Warburg effect,” by supplying lactate and other nutrients. CAFs also affect the immune system by attracting immunosuppressive cells like Tregs, MDSCs, and M2 macrophages, and by blocking the activity of CTLs and NK cells through cytokines such as IL-6, PGE2, and TGF-β [75,76].

Recent studies have shown that the tumor microenvironment, especially CAFs, plays a key role in promoting peritoneal metastasis in CRC. CAFs are found in higher numbers in peritoneal metastasis samples and have been shown to increase membrane fluidity in CRC cells, which helps them spread more easily in the peritoneal cavity [76].

Moreover, in liver metastases, CAFs often come from hepatic stellate cells and produce factors like SDF-1 and periostin, which help cancer cells survive and grow in the liver. Because of their wide range of actions and high presence in tumors, CAFs are currently being studied as possible biomarkers and treatment targets in both primary and metastatic CRC [75].

As regard TAMs, they play a key role in colorectal cancer and are a major part of the tumor microenvironment. Most TAMs in CRC are M2-polarized, meaning they support tumor growth by promoting angiogenesis, immune suppression and metastasis. In contrast, M1 macrophages have anti-tumor properties; they help to destroy tumor cells and activate strong immune responses. The balance between M1 and M2 types is influenced by local cytokines and pathways such as STAT3, STAT6, and NF-κB. In CRC, especially in low-oxygen (hypoxic) areas, M2 macrophages usually dominate, which is often linked to a worse prognosis [77,78].

Despite their general association with tumor progression, TAMs can also have anti-tumor effects, depending on where they are found in the tumor. For example, TAMs located at the invasive front of the tumor may help kill cancer cells and activate T-cells. This highlights their dual and complex role in CRC.

TAMs mainly come from circulating monocytes and can switch between M1 and M2 states. This plasticity makes them an interesting target for new treatments. Functionally, TAMs help tumors grow by supporting epithelial mesenchymal transition, releasing growth factors like VEGF and EGF, and suppressing immune responses through IL-10, TGF-β, and immune checkpoints such as PD-L1. They also influence tumor metabolism and interact with the gut microbiota, further promoting tumor survival [79].

Interestingly, some studies suggest that high levels of TAMs, along with mast cells (MCs), may be linked to better prognosis. One study on CRC tissue samples found that patients with high numbers of both TAMs and MCs had better clinical outcomes: less tumor invasion, fewer lymph nodes and distant metastases, and improved 5-year survival. Specifically, patients with high TAM or MC levels had 5-year survival rates of 65.5% and 59.3%, compared to 25.8% and 33.3% in patients with low levels. When both cell types were high, survival reached 76.2%. There was also a strong positive correlation between the amount of TAMs and MCs [78].

Overall, TAMs have a complex role in CRC. While M2-type TAMs are generally associated with worse outcomes, their impact depends on their phenotype, location, and interaction with other cells. Measuring TAM levels, especially together with mast cells, could be useful for prognosis. Their plasticity and dual role make them a potential target for future therapies.

Finally, tumor-infiltrating lymphocytes (TILs), especially CD8^+^ cytotoxic T cells, are a key part of the immune response against colorectal cancer. They tend to gather at the invasive edge of the tumor and within cancer cell clusters. High levels of intraepithelial CD8^+^ T cells are strongly linked to longer disease-free and overall survival, particularly in early-stage CRC. CD3^+^, CD8^+^, FOXP3^+^, and CD45RO^+^ T cells have also shown positive prognostic value, especially when present in the tumor center or invasive margin [80,81,82].

Microsatellite instability is associated with a higher number of TILs, especially in MSI tumors, which are often “hot” and respond better to immune checkpoint inhibitors like pembrolizumab and nivolumab. However, some mismatch repair proficient or microsatellite stable tumors with high TILs also show good prognosis, suggesting that the level of immune infiltration may be more important than MSI status alone [83,84,85].

TILs are not only linked to prognosis but may also help identify patients who will benefit most from ICIs. Studies have shown that patients with high TIL levels have higher response rates, better PFS, and longer OS when treated with immunotherapy. Because measuring tumor mutational burden can be expensive and not always available, TILs assessment may serve as a simpler and more accessible alternative biomarker in clinical practice [86,87].

TIL presence varies depending on tumor site and stage. They are more common in the primary tumor than in tumor deposits or liver metastases. As CRC progresses, TIL density tends to decrease, which may reflect the tumor’s ability to suppress or escape immune responses. In primary tumors, high TIL levels are linked to lower rates of vascular, lymphatic, and nerve invasion, and fewer lymph node metastases. This suggests a protective effect of TILs [86,88].

Importantly, TIL composition and spatial distribution should be considered alongside traditional staging systems like TNM. Despite strong evidence supporting their prognostic value, TILs are still not widely used in routine clinical assessments, partly due to variability in how they are measured and reported. However, growing support for tools like the “Immunoscore” shows a move toward integrating immune-based markers into standard cancer evaluation [89].

In summary, the tumor microenvironment in CRC plays a key role in tumor progression and prognosis. CAFs and TAMs mainly promote metastasis and immune evasion, while high TIL levels are linked to better outcomes. Integrating immune and stromal markers with standard staging could improve treatment decisions.

## 3. Conclusions and Future Directions

The omics sciences therefore significantly improve the therapeutic management of metastatic colon cancer by providing detailed and dynamic molecular information about the tumor. These data make it possible to identify specific genetic alterations and molecular profiles that guide the choice of targeted therapies, such as anti-EGFR, anti-BRAF drugs or inhibitors of other signaling pathways. In addition, integrated omics science analysis allows real-time monitoring of treatment response and early detection of the development of resistance mechanisms, enabling rapid therapeutic adjustment. This personalized approach helps clinicians decide when to intensify, modify, or discontinue a therapy, avoiding ineffective treatments and reducing patient toxicity. In this way, omics sciences support therapeutic decisions that are more precise, flexible, and tailored to the biological evolution of the tumor, improving clinical outcomes and quality of life for patients.

However, the clinical adoption of omics technologies has some major critical issues: the complexity in interpreting large volumes of data, the lack of standardization in analysis methods and interpretive parameters, the high costs that limit their dissemination and accessibility, and the biological heterogeneity of the tumor that can make it difficult to generalize results. In addition, translating omics information into concrete therapeutic recommendations requires robust clinical trials and ongoing validations. These challenges must be overcome to fully integrate omics sciences into clinical practice, but their potential in improving therapeutic decisions and patient outcomes remains very promising.

## Figures and Tables

**Table 1:** Omics sciences synthesis.

*Omics Science*	Role	Comment
***Genomics***	Identifies mutations and genetic predispositions to CRC	Traditional genomics studies have identified cancer-driving mutations (e.g., *TP53, KRAS, EGFR*) but fail to explain tumor heterogeneity.
***Epigenomics***	Reveals DNA methylation and histone modifications linked to tumor suppression or activation	
***Transcriptomics***	Examines RNA expression changes, highlighting regulatory ncRNAs like miRNAs	Transcriptomics and proteomics reveal that mRNA expression does not always correlate with protein abundance, requiring integrated analysis.
***Proteomics***	Detects CRC-related proteins and their modifications	Transcriptomics and proteomics reveal that mRNA expression does not always correlate with protein abundance, requiring integrated analysis.
***Metabolomics***	Assesses metabolic alterations in CRC cells, revealing dysbiosis associated with CRC and potential diagnostic markers	Metabolomics and microbiomics contribute to understanding cancer metabolism and tumor microenvironment interactions.
***Microbiomics***	Investigates the gut microbiota’s role	Studies integrating genomics and metabolomics have identified *Fusobacterium nucleatum* as a gut microbiome biomarker associated with CRC progression.
***Lipidomics***	Studies lipid metabolism changes as potential CRC markers	

## Data Availability

The data can be shared upon request.
